# A polyvalent phage shapes bacterial dynamics

**DOI:** 10.1128/jvi.01363-25

**Published:** 2026-05-19

**Authors:** Cristian V. Crisan, Daria Van Tyne, Joanna B. Goldberg

**Affiliations:** 1 Department of Pediatrics, Division of Pulmonary, Asthma, Cystic Fibrosis, and Sleep, Emory University School of Medicine 12239, Atlanta, Georgia, USA; 2 Emory+Children’s Center for Cystic Fibrosis and Airway Disease Research, Emory University School of Medicine 12239, Atlanta, Georgia, USA; 3 Division of Infectious Diseases, University of Pittsburgh School of Medicine 12317 , Pittsburgh, Pennsylvania, USA; Michigan State University, East Lansing, Michigan, USA

**Keywords:** polyvalent bacteriophages, bacterial dynamics, *Pseudomonas aeruginosa*, *Stenotrophomonas maltophilia*

## Abstract

Bacteria and the viruses that infect them (known as bacteriophages or phages) are important microbial ecosystem members. Antagonistic interactions between different bacteria or between bacteria and phages can profoundly impact population dynamics. Lytic phages are efficient killers that generally infect strains from a single genus or species. Polyvalent phages that target multiple unrelated hosts have been described, but their ecological significance is largely unknown. Here, we investigated how a polyvalent phage (PSA39) alters bacterial dynamics during co-cultures with susceptible hosts. *Pseudomonas aeruginosa* and *Stenotrophomonas maltophilia* are unrelated bacterial species that inhabit the same ecological niches, are often co-members of microbial ecosystems, cause similar infections, share mobile genetic elements, and can engage in complex interactions. In the presence of *P. aeruginosa* and *S. maltophilia*, PSA39 significantly reduces the recovery of both bacteria but has a stronger impact on *S. maltophilia. P. aeruginosa* adapts in the presence of PSA39, but *S. maltophilia* survival is impaired when the two bacteria are grown together and with phage. Furthermore, propagation in the presence of *S. maltophilia* cells results in higher viral titers. Both bacterial species evolve mutations in pili genes when exposed to PSA39. We propose that *P. aeruginosa*, *S. maltophilia*, and PSA39 can serve as a model system to study how polyvalent phages alter co-existing bacterial populations.

**IMPORTANCE:**

Phages are the most abundant biological entity on the planet, but polyvalent phages that infect multiple bacterial species are poorly understood. Here, we investigated how the polyvalent phage PSA39 affects two susceptible but unrelated bacterial hosts (*Pseudomonas aeruginosa* and *Stenotrophomonas maltophilia*). During co-cultures with *S. maltophilia*, *P. aeruginosa* quickly develops resistance to this virus and has an antagonistic effect on its bacterial competitor. We find that both bacterial species evolve mutations in Type IV pili genes to resist PSA39 lysis. Our study provides novel insights into the impact that polyvalent phages can have on susceptible bacteria, such as those from natural environments or from infections.

## INTRODUCTION

Bacteria live in polymicrobial environments, where they engage in antagonistic interactions with other bacteria and with viruses using an extensive weapon arsenal (1–8). Antagonistic interactions can have important effects on the structure and dynamics of microbial populations (9–13). Bacteriophages (or phages) are viruses that infect bacterial cells (14). These parasites are found in most natural and anthropogenic locations, but have also been isolated from plants, animals, and humans (14–17). Phages exhibit a high degree of morphological, genomic, and functional diversity (14, 16). Lytic phages bind to receptors on the bacterial surface, insert their genome into the cytoplasm, replicate to high numbers, and rupture cells to release virions that continue infection cycles (14). Because of their bactericidal properties, lytic phages have been successfully used in phage therapy to treat multidrug-resistant infections (18–20). By contrast, lysogenic phages integrate their genomes into bacterial chromosomes and replicate along with their hosts (21). While antibiotics can affect multiple unrelated bacteria (including those from healthy microbiomes), phages generally have a narrow host range and only infect specific bacterial genera, species, or strains (18–20).

Several studies have described polyvalent phages that can infect bacterial isolates from distinct taxonomic clades (22–26). For example, phage PEf1 infects both *Escherichia* and *Pseudomonas* species, and its infectious potential is influenced by the other bacteria present in co-cultures (22, 27). Phages DLP1 and DLP2 lyse both *Stenotrophomonas maltophilia* and *Pseudomonas aeruginosa* strains (25, 28). The ecological importance of polyvalent phages is not clear, and knowledge gaps exist about the roles played by these viruses in shaping bacterial cultures that consist of susceptible but unrelated hosts (11, 22, 27, 29).

*P. aeruginosa* and species from the *S. maltophilia* complex are opportunistic multidrug-resistant bacterial pathogens that can cause lung, blood, skin, eye, and brain infections (30–34). Immunocompromised individuals and people with conditions like cystic fibrosis (CF), chronic obstructive pulmonary disease, or cancer are especially vulnerable (31, 32, 35–38). Polymicrobial infections with *P. aeruginosa* and *S. maltophilia* can lead to worse health outcomes (33, 39–41). Both cooperative and antagonistic interactions have been observed between these pathogens (5, 42–47). *P. aeruginosa* and *S. maltophilia* can be isolated from similar natural environments (such as waters and soils) and from anthropogenic sources (like hospitals and other healthcare facilities) (31, 32, 48, 49). Furthermore, they can also share mobile genetic elements (49). However, these two species are genomically unrelated and belong to distinct taxonomic groups: *P. aeruginosa* is part of the *Pseudomonadales* bacterial order, while *S. maltophilia* is classified in the *Lysobacterales* order.

The airway microbiome of people with CF and advanced lung disease can be dominated by pathogens like *P. aeruginosa* and *S. maltophilia* (50–53). Cox et al. found that both these bacteria are abundant in the lungs of older people with CF whose airway microbiomes have few other bacterial species (53). Here, we developed a model system to study how a polyvalent phage can alter the dynamics of bacteria from low-diversity environments. Phage PSA39 was previously isolated using *P. aeruginosa* as its host. The PSA39 genome shares high similarity to the genomes of other *P. aeruginosa* phages and limited homology to the genome of a *Stenotrophomonas* phage. We observed that PSA39 also infects *S. maltophilia*, and transmission electron microscopy (TEM) imaging revealed that this phage has a *Siphoviridae*-like morphology when propagated on either *P. aeruginosa* or *S. maltophilia*. Over longer timeframes, *P. aeruginosa* adapts in the presence of *S. maltophilia* and PSA39, while *S. maltophilia* survival is significantly impaired when co-cultured with *P. aeruginosa* and this phage. Both *P. aeruginosa* and *S. maltophilia* evolve resistance to PSA39 by acquiring mutations in pili genes. Our results provide insights into the impact that polyvalent phages can have on bacterial dynamics.

## MATERIALS AND METHODS

### Bacterial strains and growth conditions

*Stenotrophomonas maltophilia* strains were streaked on LB plates supplemented with imipenem (20 µg/mL), *Pseudomonas aeruginosa* and *Burkholderia cenocepacia* strains were streaked on *P. aeruginosa* isolation agar, *Staphylococcus aureus* was streaked on *Staphylococcus* isolation agar (trypticase soy agar with 7.5% NaCl), and *Escherichia coli* was streaked on LB. Single colonies of the indicated bacterial strains were inoculated in liquid LB and incubated at 37°C. All bacterial strains used in this study are listed in Table S1.

### Bacterial phage susceptibility

**Fig 1:**
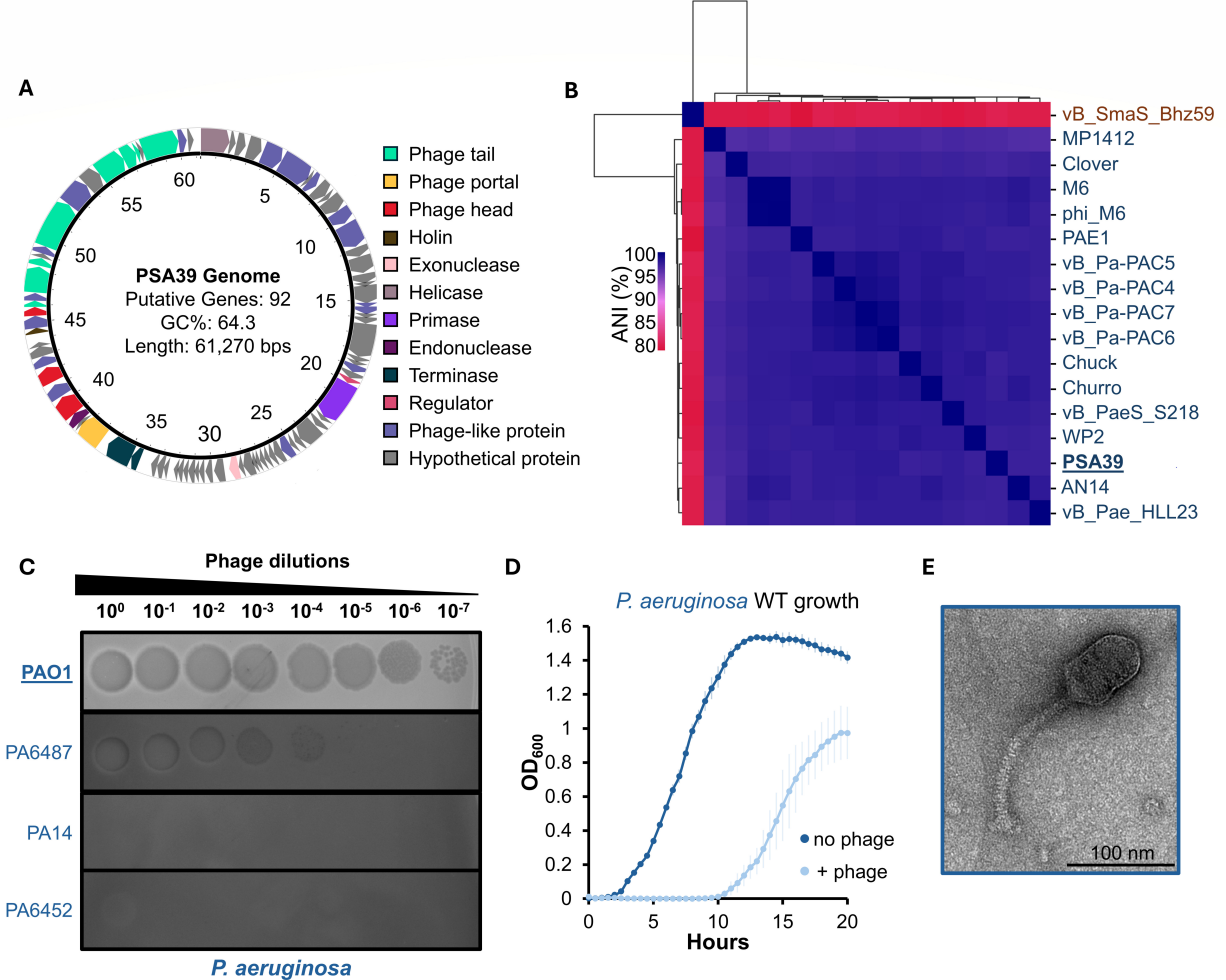
PSA39 infects some *P. aeruginosa* strains and has a *Siphoviridae-*like morphology. (**A**) PSA39 viral genomic DNA was extracted following growth with *P. aeruginosa* PAO1 and sequenced using both Illumina and Oxford Nanopore technologies. The phage genome was annotated using PHASTEST and Pharokka (58, 59). Numbers inside the circle represent nucleotide positions in kbp. (**B**) The PSA39 genome was used to perform a blastn search. The top 15 *P. aeruginosa* phage genomes (based on sequence identity) and the vB_smaS_Bhz59 *Stenotrophomonas* phage genome were used to construct an average nucleotide identity (ANI) matrix. (**C**) PSA39 lysate was serially diluted, and 2 µL of each dilution was spotted on the indicated *P. aeruginosa* strains. Results are representative of three biological replicates (*N* = 3). (**D**) Overnight *P. aeruginosa* cultures were back-diluted 1:50 in fresh liquid LB, grown at 37°C for 3 hours, and adjusted to an OD_600_ of 0.1. One hundred microliters of bacteria and 5 µL of PSA39 phage (~3 × 10^9^ virions as estimated from plaques formed on *P. aeruginosa* lawns) were added as indicated to 3 mL of LB. Two hundred microliters of each culture was transferred to a 96-well plate, and OD_600_ values were recorded at 37°C. Four biological replicates (*N* = 4) were used to calculate averages. Error bars represent standard deviation. (**E**) Phages were propagated on *P. aeruginosa*, and lysates were spotted on carbon-coated copper grids, stained with 2% uranyl acetate, and imaged with a Hitachi HT7800 120 kV Transmission Electron Microscope at 60,000× magnification.

**Fig 2:**
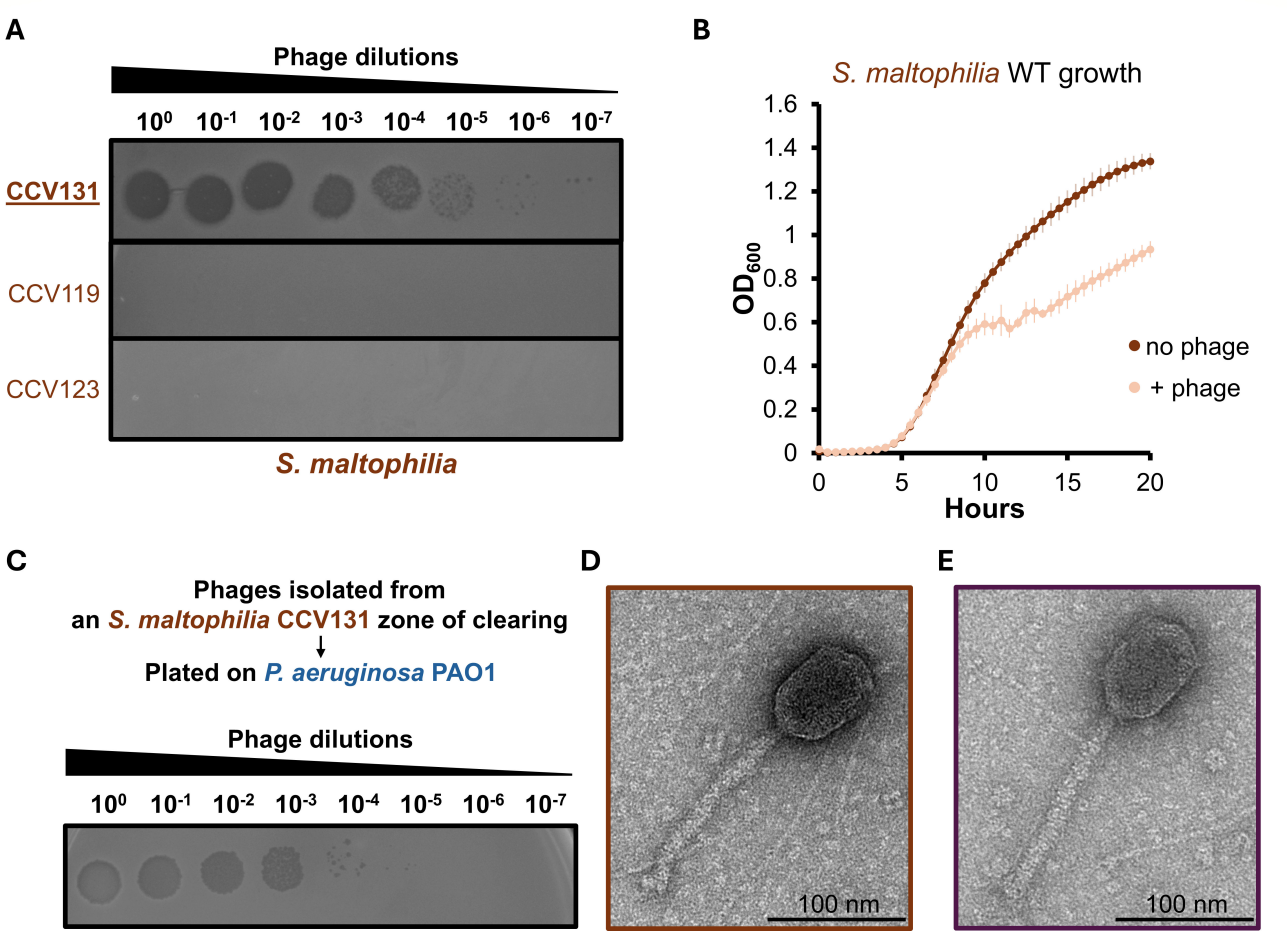
PSA39 lyses some *S. maltophilia* strains and retains a *Siphoviridae*-like morphology when propagated on this bacterium*.* (**A**) PSA39 lysate was serially diluted, and 2 µL of each dilution was spotted on the indicated *S. maltophilia* strains. Results are representative of three biological replicates (*N* = 3). (**B**) Overnight *S. maltophilia* cultures were back-diluted 1:50 in fresh liquid LB, grown at 37°C for 3 hours, and adjusted to an OD_600_ of 0.1. One hundred microliters of bacteria and 5 µL of PSA39 phage (~3 × 10^9^ virions as estimated from plaques formed on *P. aeruginosa* lawns) were added as indicated to 3 mL of LB. Two hundred microliters of each culture was transferred to a 96-well plate, and OD_600_ values were recorded at 37°C. Four biological replicates (*N* = 4) were used to calculate averages. Error bars represent standard deviation. (**C**) Phages were harvested from an *S. maltophilia* plaque, resuspended in sterile PBS, serially diluted, and 2 µL of each dilution was spotted on *P. aeruginosa* PAO1. Results are representative of three biological replicates (*N* = 3). Phages were propagated on *S. maltophilia* alone (**D**) or *S. maltophilia* with *P. aeruginosa* co-cultures (**E**), and lysates were spotted on carbon-coated copper grids, stained with 2% uranyl acetate, and imaged with a Hitachi HT7800 120 kV Transmission Electron Microscope at 60,000× magnification.

To determine the susceptibility of strains to PSA39 (Fig. 1C and 2A), individual colonies of the indicated bacteria were inoculated in 3 mL of liquid LB, grown overnight at 37°C, diluted 1:50 in fresh media, and incubated at 37°C with shaking. After 3 hours, ~200 µL of bacterial culture was mixed with 3 mL of pre-heated soft LB agar (0.7% agar) and distributed on LB plates. PSA39 phage lysates were serially diluted in LB, and 2 µL of each dilution was spotted on bacterial lawns. Plates were imaged after overnight incubation at 37°C. For growth curve experiments in Fig. 1D and 2B, bacteria from overnight cultures grown in liquid LB were diluted 1:50 in fresh media and incubated at 37°C with shaking. After 3 hours, cultures were set to an OD_600_ of 0.1. One hundred microliters of the indicated bacteria (at an OD_600_ of 0.1) and 5 µL of PSA39 phage were added to 3 mL of liquid LB. A 200 µL aliquot of this mixture was added to a 96-well plate and incubated with continuous shaking at 37°C. OD_600_ readings were recorded using a BioTek Synergy H1 Plate Reader.

### Twenty-hour evolution experiments

Single colonies of the indicated bacterial strains were each inoculated separately in 3 mL of liquid LB, grown overnight at 37°C, diluted 1:50 in fresh media, and incubated at 37°C with shaking. After 3 hours, cultures were set to an OD_600_ of 0.1. Cells were vortexed briefly, and 100 µL of the indicated bacterial ratios at an OD_600_ of 0.1 and 5 µL of PSA39 phage (~3 × 10^9^ virions as estimated from plaques formed on *P. aeruginosa* lawns) were added to 3 mL of liquid LB, where indicated. Cultures were incubated at 37°C with shaking (200 rpm) for the indicated times, serially diluted, and 5 µL of each dilution was spread on cetrimide agar (to select for *P. aeruginosa*) or LB plates supplemented with imipenem (20 µg/mL, to select for *S. maltophilia*). Colonies were counted after overnight incubation at 37°C.

To determine plaque-forming units (PFUs) after propagation with the indicated bacterial strains, liquid cultures with PSA39 were centrifuged at 4,000 × *g* for 30 minutes at 25°C, and supernatants were filtered with 0.22 µm filters. Phage lysates were stored at 4°C. Host bacterial cells were harvested from plates following overnight growth, resuspended in LB, set to an OD_600_ of 0.1 in 3 mL of liquid LB, and incubated at 37°C with shaking. After 2 hours, ~200 µL of bacterial cultures was mixed with 3 mL of pre-heated soft LB agar (0.7% agar) and distributed on LB plates. Phage lysates from the indicated experiments were serially diluted in LB, and 2 µL of each dilution was spotted on bacterial lawns. Plaques were counted after overnight incubation at 37°C.

### Three-day evolution experiments

For 3-day polymicrobial culture experiments, cultures were started as described above, and 1:100 dilutions were made each day into fresh 3 mL of liquid LB. Each day, bacterial CFUs and viral PFUs were determined as described above.

Bacteria resistant to PSA39 were isolated after 1 day of exposure to phage. After 24 hours of growth at 37°C in 3 mL of LB as described above in the presence or absence of PSA39, cultures of bacteria originating from three separate, individual colonies were serially diluted and spotted on LB plates supplemented with imipenem (for *S. maltophilia*) or cetrimide (for *P. aeruginosa*). After overnight growth, three individual colonies from cultures grown in the presence or absence of PSA39 were streaked again on LB plates supplemented with imipenem (for *S. maltophilia*) or cetrimide (for *P. aeruginosa*) and grown overnight at 37°C. Bacteria were harvested from plates to make glycerol freezer stocks.

### Bacterial genome sequencing

Bacteria from *S. maltophilia* and *P. aeruginosa* freezer stocks were streaked on LB plates supplemented with imipenem (for *S. maltophilia*) or cetrimide (for *P. aeruginosa*). Following overnight growth, bacteria were harvested from plates and resuspended into 500 µL of sterile PBS. Samples were centrifuged at 5,000 × *g* for 5 minutes, the supernatant was discarded, and cell pellets were frozen in a dry ice ethanol bath. Genomes were extracted using bead-beating cellular lysis and sequenced at SeqCoast Genomics (https://seqcoast.com/). DNA samples were prepared for sequencing using the Illumina DNA Prep Tagmentation Kit (#20060059) with Illumina Unique Dual Indexes. An Illumina NextSeq 2000 platform with a 300-cycle flow cell kit was used for sequencing to produce 2 × 150 bp paired reads. Optimal base calling was supported by spiking a 1%–2% PhiX (accession number: NC_001422) control. DRAGEN v4.2.7 was used for read demultiplexing, trimming, and analytics. Mutations were identified using breseq v0.35.5 (54) and were confirmed using Oxford Nanopore sequencing.

### PSA39 phage propagation, genome extraction, and sequencing

A colony of *P. aeruginosa* PAO1 was inoculated in 20 mL of LB and incubated at 37°C with shaking. When the culture reached an OD_600_ of ≈0.5, 200 µL of PSA39 phage lysate was added. Following overnight incubation at 37°C, the culture was centrifuged at 25°C and 4,000 × *g* for 30 minutes. Supernatants were filtered twice using 0.22 µm filters. Three milliliters of PSA39 lysate was added to an Amicon 4 mL 100 kDa cutoff filter and centrifuged for 20 minutes at 3,000 × *g* and 25°C. The flowthrough was discarded, and ~100 µL of concentrated lysate was obtained. Four hundred microliters of sterile PBS, 50 µL of Turbo DNase buffer (Thermo), 1 µL of Turbo DNase (Thermo, 2 U/µL), and 3 µL of RNase A (Promega, 4 mg/mL) were added and incubated at 37°C for 90 minutes. Twenty microliters of a 0.5 M EDTA solution and 1.25 µL of Proteinase K (20 mg/mL) were added and incubated at 56°C for 90 minutes. Five hundred microliters of AL buffer from the Qiagen DNeasy Blood & Tissue Kit was added to the solution and mixed thoroughly by inverting the tube multiple times. The sample was incubated at 80°C for 20 minutes, and 500 µL of 100% ethanol was added. The mixture was transferred to a Qiagen DNeasy Mini spin column and centrifuged for 1 minute at 6,000 × *g*. The flow-through was discarded. Five hundred microliters of AW1 buffer was added, and the column was centrifuged for 1 minute at 6,000 × *g*. The flow-through was again discarded, 500 µL of AW2 buffer was added, and the column was centrifuged for 3 minutes at 20,000 × *g*. DNA was eluted in 30 µL of buffer AE. The sample was diluted to ~80 ng/µL and sent for Oxford Nanopore and Illumina hybrid sequencing at Plasmidsaurus (https://plasmidsaurus.com/).

For Oxford Nanopore sequencing, an amplification-free long-read sequencing library was constructed using the v14 library prep chemistry (Rapid Barcoding Kit 96 V14) and sequenced using a PromethION P24 instrument with R10.4.1 flow cells. The bottom 5% worst fastq reads were removed with Filtlong v0.2.1 (default parameters, available at https://github.com/rrwick/Filtlong), and reads were downsampled to 250 Mb to create an assembly sketch with Miniasm v0.3 (55). Reads were re-downsampled to ~100× coverage with heavy weight applied to remove low-quality reads. Adapters were trimmed using MinKnow. Reads were assembled using Flye v2.9.1 with parameters selected for high quality ONT reads and polished with Medaka v1.8.0 (available at https://github.com/nanoporetech/medaka) (56).

For Illumina sequencing, libraries were constructed using the Illumina DNA Prep Kit and sequenced on a NextSeq2000 instrument with paired-end 2 × 150 bp reads. Illumina reads were used to polish the Oxford Nanopore long-read genome using Polypolish v0.6.0 (57). The PSA39 genome was annotated using PHASTEST v3.0 and Pharokka v1.3.2 with default parameters (58, 59). Illumina reads obtained from the sequencing of the PSA39 genome were analyzed using metaSPAdes v3.15.3 to confirm sample purity (60). PhaBOX v2.0 was used for phage classification (61).

### Average nucleotide identity and genome comparison analyses

A blastn search was conducted in April 2025 using the PSA39 genome as the input and the Core nucleotide (core_nt) database (62). Results with <30% coverage were excluded from the analysis. The top 15 *P. aeruginosa* phage genomes (based on percent identity to PSA39) and *Stenotrophomonas* phage vB_SmaS_Bhz59 were retrieved and used to build an ANI matrix using ANIclustermap v1.2.0 (available at https://github.com/moshi4/ANIclustermap).

### Type IV pili (T4P) protein amino acid homology comparisons

The amino acid sequences of the indicated *P. aeruginosa* PAO1 T4P proteins were used as queries to perform blastp (BLAST+ v2.17.0) searches with default parameters (BLOSUM62 matrix, gap cost existence: 11, gap cost extension: 1) against all *S. maltophilia* CCV131 annotated proteins. The query coverage and percentage identity for each *S. maltophilia* CCV131 protein homolog with the highest E value are displayed in Table S2.

### TEM imaging

Bacteria were harvested from plates, resuspended in LB, set to an OD_600_ of 0.1, and incubated at 37°C with shaking. After 2 hours, 50 µL of PSA39 phage was added to a final volume of 1 mL bacterial culture (*P. aeruginosa* alone, *S. maltophilia* alone, or *S. maltophilia* and *P. aeruginosa* at a 1:1 ratio). Following overnight incubation at 37°C with shaking, cultures were centrifuged for 30 minutes at 4,000 × *g*, and supernatants were filtered twice using 0.22 µm filters. Phages were imaged at the University of Maryland Keith R. Porter Imaging Facility (https://kpif.umbc.edu/bacteriophage-imaging/). Ten microliters of crude phage lysate was added to 200 mesh formvar-covered, carbon-coated copper grids (EMS, Hatfield, PA, USA). After 1 minute of incubation, grids were briefly rinsed with ultra-pure water and stained with 2% uranyl acetate for 2 minutes. Images were acquired at 100 kV and 60,000× magnification using a Hitachi HT7800 120 kV TEM equipped with an AMT Nanosprint15 B digital camera. Five random phage particles from different fields of view were used for capsid length, capsid width, and tail length measurements.

### Statistical analyses

For all statistical analyses, ANOVA with *post hoc* Tukey HSD tests were performed in JASP v0.95.3 to compare the means of each group to every other group and to determine statistical significance (63). For Fig. 2A and B, bacterial ratios from each treatment and the presence/absence of phage were used as fixed factors, while CFUs were used as the dependent variable. For Fig. 2C and D, bacterial ratios from each treatment were used as fixed factors, while PFUs were used as the dependent variables. Replicate numbers (*N*) for each experiment are indicated in the figure legends. All *P* value results from ANOVA with *post hoc* Tukey HSD tests are listed in Tables S3 to S6.

## RESULTS

### PSA39 is a *Yuavirus* DNA phage that infects both *P. aeruginosa* and *S. maltophilia*

Phage PSA39 was isolated from wastewater effluent in Pittsburgh (Pennsylvania, USA) using a *P. aeruginosa* host (64). The complete viral genome is 61,270 bp in length, encodes 86 putative phage genes (92 total putative genes), and has a GC% of ≈64.3 (Fig. 1A). Based on its sequence, PSA39 is predicted to belong to the *Yuavirus* genus (61, 65). It shares high similarity with multiple *P. aeruginosa* phages and limited similarity to a previously sequenced *Stenotrophomonas* phage (Fig. 1B).

PSA39 forms clear plaques when spotted on the *P. aeruginosa* PAO1 lab strain and on the corneal infection isolate PA6487 (Fig. 1C; Fig. S1A and B) (66). By contrast, it is unable to form plaques on the *P. aeruginosa* PA14 lab strain or on the PA6452 corneal isolate (Fig. 1C) (66). When grown in 96-well plate liquid cultures without phage, *P. aeruginosa* PAO1 enters exponential growth after ~2–3 hours. In the presence of PSA39, *P. aeruginosa* PAO1 growth is delayed until ~10–12 hours (Fig. 1D). Transmission electron microscopy imaging revealed that PSA39 has a *Siphoviridae*-like morphology (Fig. 1E).

Since PSA39 shares limited homology to the *Stenotrophomonas* phage vB_SmaS_Bhz59, we hypothesized that it might also infect strains from this bacterial genus. We found that PSA39 can form plaques on the *Stenotrophomonas maltophilia* complex strain CCV131 but cannot infect CCV119 or CCV123 (Fig. 2A; Fig. S1C). Using this assay, the plaquing efficiency of PSA39 (propagated on *P. aeruginosa* PAO1) was reduced by ~100-fold when plated on *S. maltophilia* CCV131 compared to *P. aeruginosa* PAO1 (Fig. 1C and 2A). Furthermore, PSA39 does not form plaques on *Staphylococcus aureus* JE2, *Burkholderia cenocepacia* K56-2, or *Escherichia coli* DH5α (Fig. S2). In contrast to *P. aeruginosa* PAO1, the time of entry into exponential phase for *S. maltophilia* CCV131 is not affected by PSA39, but growth is impaired after bacteria reach mid-log phase (Fig. 2B). Since phage stocks were created following propagation on *P. aeruginosa* PAO1, we sought to confirm that plaques or zones of clearing formed on *S. maltophilia* strains are due to PSA39 and not due to *P. aeruginosa* secreted factors with antibacterial properties (67). As predicted, filtered supernatant obtained from *P. aeruginosa* cultures grown without PSA39 does not form plaques or clear zones on *S. maltophilia* (Fig. S3).

To further confirm that PSA39 can lyse both strains, we harvested phages directly from zones of clearing formed on *S. maltophilia* CCV131 and spotted them onto a *P. aeruginosa* PAO1 bacterial lawn (Fig. 2C). We observed that phages harvested from *S. maltophilia* CCV131 zones of clearing retain their ability to form plaques on *P. aeruginosa* PAO1 (Fig. 2C). PSA39 has the same *Siphoviridae-*like morphology, and similar capsid length, capsid width, and tail length when infecting *P. aeruginosa* PAO1 alone, *S. maltophilia* CCV131 alone, or *S. maltophilia*/*P. aeruginosa* co-cultures (Fig. 2D and E; Fig. S4). These results provide evidence that PSA39 is a *Yuavirus* phage that can infect both *P. aeruginosa* PAO1 and *S. maltophilia* CCV131.

### PSA39 alters dynamics of *P. aeruginosa* and *S. maltophilia* co-cultures

**Fig 3:**
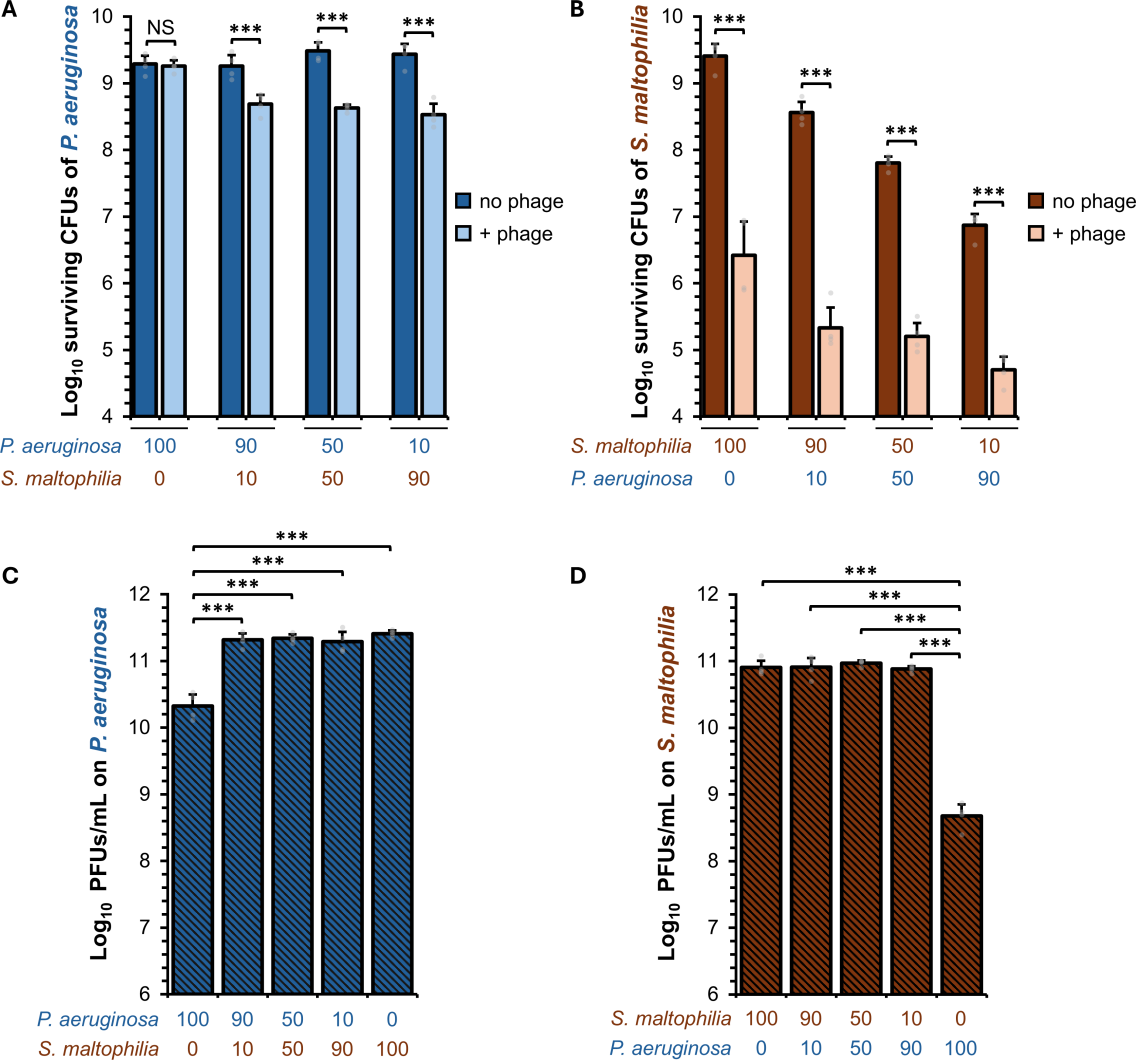
Recovery of *P. aeruginosa* and *S. maltophilia* during co-cultures with both bacteria is affected by PSA39. Overnight cultures of *P. aeruginosa* and *S. maltophilia* were back-diluted 1:50 in fresh liquid LB, grown at 37°C for 3 hours, and adjusted to an OD_600_ of 0.1. One hundred microliters of bacteria at the indicated ratios were added to 3 mL of liquid LB. Five microliters of PSA39 phage (~3 × 10^9^ virions as estimated from plaques formed on *P. aeruginosa* lawns) was also added where indicated. Cultures were grown with shaking at 37°C. After 20 hours, cells were serially diluted and plated on cetrimide plates to select for *P. aeruginosa* (**A**) or LB plates supplemented with imipenem (20 µg/mL) to select for *S. maltophilia* (**B**). CFUs were counted following overnight incubation at 37°C. Liquid cultures were also centrifuged and filtered to obtain phage lysates, which were then serially diluted and spotted on bacterial lawns of *P. aeruginosa* (**C**) or *S. maltophilia* (**D**). PFUs were counted following overnight incubation at 37°C. Four biological replicates (*N* = 4) were used to determine CFUs and PFUs averages. Error bars represent standard deviation. Circles represent individual replicates. ANOVA with *post hoc* Tukey tests was used to determine statistical significance. ***P < 0.001.

We next sought to determine how PSA39 affects *P. aeruginosa* PAO1 (referred to as *P. aeruginosa* henceforth) and *S. maltophilia* CCV131 (referred to as *S. maltophilia* henceforth) recovery when the two bacteria are grown together. We performed co-cultures at different inoculation ratios in the presence or absence of PSA39 and determined the survival of both bacteria after 20 hours (Fig. 3A and B). In the absence of phage, *P. aeruginosa* recovery is not affected by *S. maltophilia*, but *S. maltophilia* recovery is reduced by *P. aeruginosa* in a dose-dependent manner (Fig. 3A and B). In the presence of phage, recovery of *P. aeruginosa* is reduced during co-culture with *S. maltophilia* but not during monoculture (Fig. 3A). Recovery of *S. maltophilia* is further diminished by PSA39 when co-cultured with *P. aeruginosa* (Fig. 3B). These findings indicate that both *P. aeruginosa* and *S. maltophilia* recovery is negatively impacted by PSA39 during co-culture.

To estimate PSA39 titers when propagated on *P. aeruginosa* alone, on *S. maltophilia* alone, or on both bacteria, we determined the number of PFUs obtained by spotting phage lysates on *P. aeruginosa* or *S. maltophilia* bacterial lawns (Fig. 3C and D). Phage titers from all cultures with *S. maltophilia* are significantly higher compared to titers from cultures without *S. maltophilia* (Fig. 3C and D). PSA39 propagated in the presence of *S. maltophilia* also has similar plaquing efficiency when plated on either *P. aeruginosa* or *S. maltophilia* (Fig. 3C and D). These results suggest that *S. maltophilia* allows PSA39 to replicate to higher titers in liquid cultures compared to *P. aeruginosa*.

### Growth with *P. aeruginosa* over time hinders *S. maltophilia* recovery in the presence of PSA39

**Fig 4:**
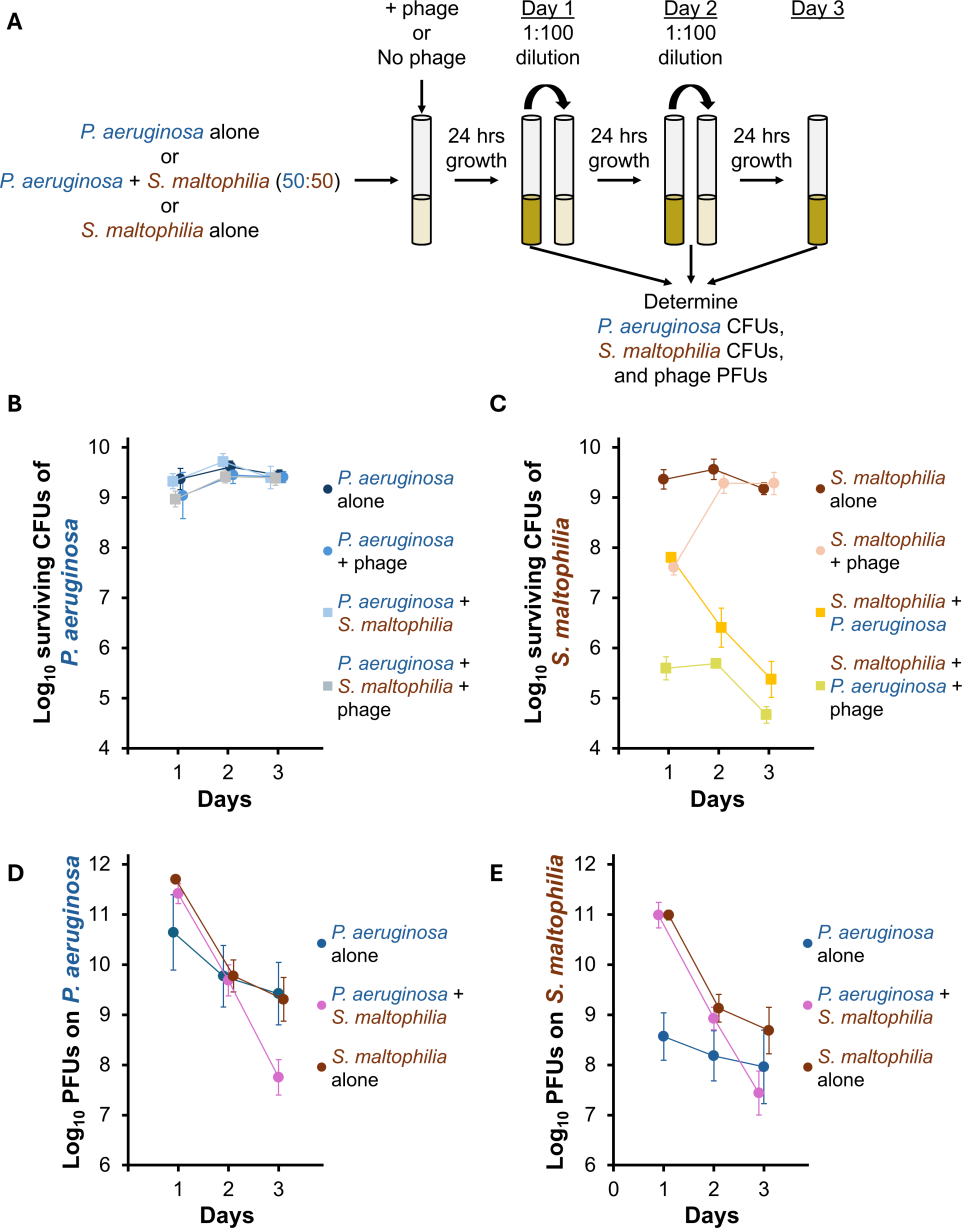
*P. aeruginosa* recovers during co-culture with *S. maltophilia* in the presence of PSA39 and impairs *S. maltophilia* recovery*.* At the beginning of the experiment (day 0), overnight cultures of *P. aeruginosa* and *S. maltophilia* were back-diluted 1:50 in fresh liquid LB, grown at 37°C for 3 hours, and adjusted to an OD_600_ of 0.1. One hundred microliters of bacteria at the indicated ratios were added to 3 mL of liquid LB. The number of CFUs per milliliter was not measured at the beginning of the experiment (T0). Five microliters of PSA39 phage (~3 × 10^9^ virions as estimated from plaques formed on *P. aeruginosa* lawns) was also added where indicated (**A**). Cultures were grown with shaking at 37°C. Every day, 1:100 dilutions of the cultures were made into fresh LB tubes. Cells were serially diluted and plated on cetrimide plates to select for *P. aeruginosa* (**B**) or LB plates supplemented with imipenem (20 µg/mL) to select for *S. maltophilia* (**C**). CFUs were counted following overnight incubation at 37°C. Liquid cultures were also centrifuged and filtered to obtain phage lysates, which were then serially diluted and spotted on bacterial lawns of *P. aeruginosa* (**D**) or *S. maltophilia* (**E**). PFUs were counted following overnight incubation at 37°C. Six biological replicates (*N* = 6) were used to determine PFUs and CFUs averages for all conditions except for *P. aeruginosa* alone + PSA39 CFUs on day 1, where five biological replicates (*N* = 5) were used. Error bars represent standard deviation.

To observe how PSA39 affects polymicrobial cultures over longer periods of time, we monitored the survival of each bacterial species (alone or at 1:1 co-culture ratios) in the absence or presence of phage following daily transfers into fresh growth media (Fig. 4A). In the absence of PSA39, *P. aeruginosa* recovery is unaffected by *S. maltophilia*, but *S. maltophilia* abundance is progressively reduced each day during co-culture with *P. aeruginosa* (Fig. 4B and C). PSA39 does not affect the recovery of *P. aeruginosa* after 3 days, even when *S. maltophilia* is present (Fig. 4B). After 3 days, *S. maltophilia* recovery is not influenced by PSA39 during monoculture, but the presence of this phage further diminishes *S. maltophilia* recovery during co-culture with *P. aeruginosa* (Fig. 4C).

To determine how viral titers change over time, we spotted phage dilutions from each condition onto *P. aeruginosa* and *S. maltophilia* bacterial lawns (Fig. 4A). PFUs decrease daily following incubation with *P. aeruginosa* alone, *S. maltophilia* alone, or co-culture with both bacterial species (Fig. 4D and E). Overall, these observations indicate that the growth of *S. maltophilia* in the presence of *P. aeruginosa* negatively impacts its ability to recover from PSA39 infection, while viral titers are progressively reduced during the experimental timeframe.

### *P. aeruginosa* and *S. maltophilia* strains resistant to PSA39 have mutations in Type IV pili genes

**Fig 5:**
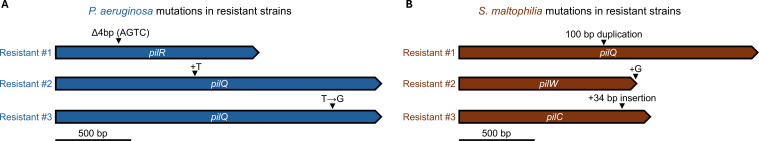
*P. aeruginosa* and *S. maltophilia* strains resistant to PSA39 have mutations in Type IV pili genes*.* Illumina and Oxford Nanopore whole-genome sequencing was performed on three resistant *P. aeruginosa* (**A**) and *S. maltophilia* (**B**) strains, and mutations were identified using breseq (54). Mutation types and locations are displayed for pili genes in *P. aeruginosa* (blue) and *S. maltophilia* (red).

We isolated and sequenced *P. aeruginosa* and *S. maltophilia* colonies (three from each species) after growth in liquid culture with or without PSA39. We confirmed that isolates recovered following growth in the presence of phage are resistant to PSA39 (Fig. S5). All *P. aeruginosa* and *S. maltophilia* strains evolved in the presence of PSA39 harbor mutations in Type IV pili (T4P) genes (Fig. 5A and B) (68, 69). One resistant *P. aeruginosa* strain has a 4-nucleotide deletion in the gene coding for the PilR transcriptional activator (70), while the other two resistant *P. aeruginosa* strains harbor a nonsynonymous G → T substitution and a +T insertion, respectively, in the *pilQ* gene (Fig. 5A) (71). Resistant *S. maltophilia* strains have mutations in *pilW* (a +G insertion after the “T” nucleotide in the “TGA” stop codon), *pilC* (a 34-nucleotide insertion), and *pilQ* (a 100-nucleotide duplication) (72, 73) (Fig. 5B). The +G insertion in *pilW* disrupted the stop codon and resulted in the addition of 68 amino acids at the encoded protein’s C-terminus. *P. aeruginosa* or *S. maltophilia* strains evolved without PSA39 lack mutations in known T4P genes.

## DISCUSSION

While living in natural environments and during chronic infections, bacteria and phages are engaged in constant competitions (3). The ability of phages to infect multiple bacterial species could have important benefits, especially during events that selectively eliminate only some hosts (e.g. treatment with narrow-spectrum antibiotics). It is possible that many phages evolved to infect multiple bacterial hosts, but this ability is largely understudied (11, 22). Furthermore, these polyvalent viruses are likely more prevalent than currently appreciated (11, 22). The presence of diverse bacterial members can modulate the dynamics of communities containing phages known to target a single bacterial species (74–80). However, the effects that polyvalent phages have on susceptible but unrelated hosts during co-cultures remain poorly understood (22, 23, 26, 28, 78).

In this study, we observed that the polyvalent phage PSA39 infects both *P. aeruginosa* and *S. maltophilia* strains. We find that during co-cultures in the presence of PSA39, *P. aeruginosa* recovers faster than *S. maltophilia* and hinders the growth of its competitor bacteria. Although *P. aeruginosa* and *S. maltophilia* are taxonomically unrelated, the two bacteria are found in the same ecological niches and cause polymicrobial infections from which they are often co-isolated (31–33). We hypothesize that a PSA39 viral ancestor may have been exposed to both *P. aeruginosa* and *S. maltophilia* and evolved to infect both species. Even though we observe that PSA39 has lytic properties, it is possible that this phage may also display lysogenic behavior.

Previous studies have described antagonistic and cooperative interactions between *P. aeruginosa* and *S. maltophilia* (5, 42–47). These interactions are likely strain-specific and are influenced by experimental conditions (42, 44, 46, 81). Both *P. aeruginosa* and *S. maltophilia* harbor an extensive arsenal of antibacterial weapons and can become the dominant species in microbiomes, such as those from the airways of people with CF (4, 5, 43–45, 51–53, 81). Pyocyanin, a toxic compound secreted by *P. aeruginosa*, has antibacterial properties against *S. maltophilia* (82). We observe that the polyvalent phage PSA39 amplifies the antagonistic effect that *P. aeruginosa* has on the recovery of *S. maltophilia*. Apparent competition, which occurs when two unrelated species are preyed upon by the same predator, could provide an explanation for the additive antagonistic effect observed when the polyvalent phage was present in a co-culture with both susceptible bacteria (83–86). The presence of a heterologous bacterium provides a replicative host for the phage, which can then replicate to higher titers and infect the other competitor bacterium.

*P. aeruginosa* and *S. maltophilia* strains evolved in the presence of PSA39 harbor mutations in Type IV pili (T4P) genes. T4P are thin, hair-like protrusions on the bacterial surface that contribute to multiple behaviors like motility, adhesion, and virulence (68, 69). These bacterial structures are also common phage receptors (87). *P. aeruginosa* strains resistant to PSA39 have disruptions in genes encoding PilR (a response regulator that has been proposed to activate transcription of T4P genes) (70) and PilQ (an outer membrane secretin required for T4P assembly and function) (88). Similarly, *S. maltophilia* strains resistant to PSA39 evolved mutations in genes encoding PilQ, PilC (an essential T4P membrane protein), and PilW (predicted to be part of the T4P structure) (71–73). These findings provide evidence that PSA39 uses T4P from both *P. aeruginosa* and *S. maltophilia* as receptors to infect cells. Phages DLP1 and DLP2 also use T4P as receptors to infect and lyse both *P. aeruginosa* and *S. maltophilia* (28). However, these phages’ genomes do not share homology to the PSA39 genome; while DLP1 and DLP2 are predicted to belong to the *Septimatrevirus* genus, PSA39 has homology to viruses from the *Yuavirus* genus (28, 61).

Some *P. aeruginosa* and *S. maltophilia* strains tested here are intrinsically resistant to PSA39 lysis. Since we determined that T4P are likely receptors for this phage, it is possible that the structure and/or regulation of pili proteins in these “resistant” strains prevent infection. We observed that *P. aeruginosa* PAO1 T4P proteins and their *S. maltophilia* CCV131 homologs share limited amino acid similarity (Table S2). Among all proteins, the average identity between homologs from the two bacterial species is ~47%. The highest identities are ~83% for PilG (predicted to be a T4P regulator in *P. aeruginosa*) and ~79% for PilT (predicted to form the *P. aeruginosa* T4P retraction motor) (68, 69). PilA, which is the major T4 pilin, has ~43% identity (68, 69). It is possible that PSA39 binds a conserved region from a *P. aeruginosa* PAO1 and *S. maltophilia* CCV131 T4P protein. Other cellular components, such as lipopolysaccharides, membrane proteins, or antiphage defense systems, could also be important in determining resistance to PSA39.

In conclusion, we demonstrate that a polyvalent phage alters the dynamics of bacterial cultures with two susceptible hosts. Both *P. aeruginosa* and *S. maltophilia* can colonize and dominate microbial environments that have a low bacterial diversity, such as those from the airways of people with CF. We propose that these two bacteria and PSA39 can serve as model systems to examine the effects that polyvalent phages have on microbial dynamics. Future work will determine how external conditions affect bacterial responses to PSA39, how antiphage immune systems influence susceptibility, and how this virus adapts in the presence of different bacterial hosts.

## Data Availability

Sequence data are available in NCBI under accession numbers MZ089741 and PRJNA1258269.

## SUPPLEMENTAL MATERIAL

Supplemental figures jvi.01363-25-s0001.docx Figures S1 to S5.

Supplemental tables jvi.01363-25-s0002.docx Tables S1 to S6.

ASM does not own the copyrights to Supplemental Material that may be linked to, or accessed through, an article. The authors have granted ASM a non-exclusive, world-wide license to publish the Supplemental Material files. Please contact the corresponding author directly for reuse.
