# Supplementary material for: A polyvalent phage shapes bacterial dynamics
Source: J Virol. 2026 May 19;100(6):e01363-25. doi: 10.1128/jvi.01363-25 (PMC13288487; doi:10.1128/jvi.01363-25)
Supplement: Supplemental figures — Figures S1 to S5. [file jvi.01363-25-s0001.docx]

**A Polyvalent Phage Shapes Bacterial Dynamics**

**Supplemental Figures**

Cristian V. Crisan^1,2^, Daria Van Tyne^3^, and Joanna B. Goldberg^1,2*^

^1^ Department of Pediatrics, Division of Pulmonary, Asthma, Cystic Fibrosis, and Sleep, Emory University School of Medicine, Atlanta, Georgia, USA.

^2^ Emory+Children's Center for Cystic Fibrosis and Airway Disease Research, Emory University School of Medicine, Atlanta, Georgia, USA.

^3^ Division of Infectious Diseases, University of Pittsburgh School of Medicine, Pittsburgh, Pennsylvania, USA.

*Corresponding author email: [joanna.goldberg@emory.edu](mailto:joanna.goldberg@emory.edu)

Corresponding author address: Department of Pediatrics, Emory University School of Medicine, O. Wayne Rollins Research Center, 1510 Clifton Road NE, Suite 3009, Atlanta, Georgia 30322, USA


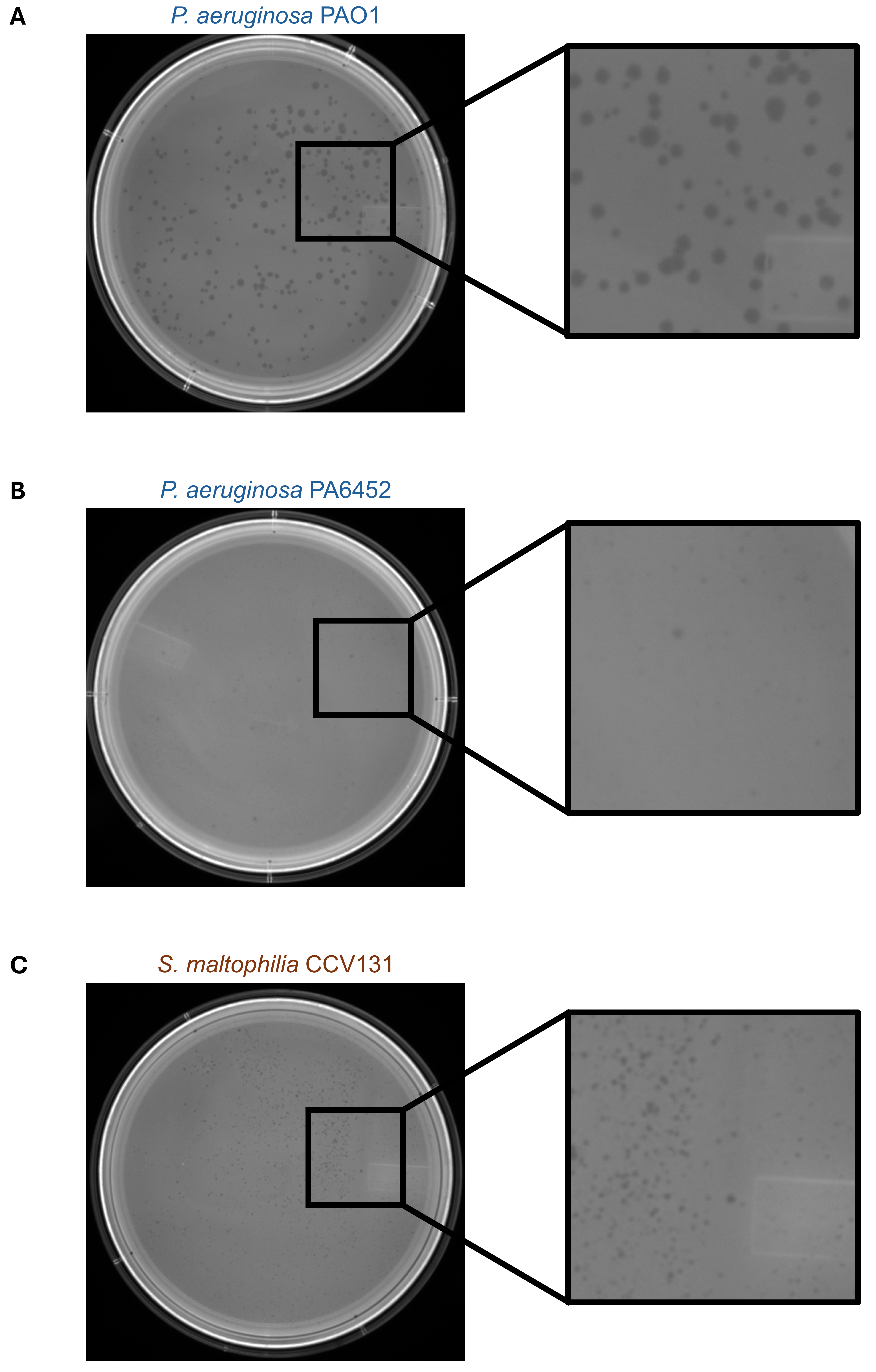


**Supplemental Figure 1. PSA39 forms plaques on *P. aeruginosa* PAO1, *P. aeruginosa* PA6452, and *S. maltophilia* CCV131.** Host bacterial cells were harvested from plates following overnight growth, resuspended in LB, set to an OD_600_ = 0.1 in 3 mL of liquid LB, and incubated at 37°C with shaking. After two hours, ~200 µL of bacterial cultures were mixed with 3 mL of pre-heated soft LB agar (0.7% agar). PSA39 phage lysate was serially diluted and 100 µL of PSA39 lysate from the 10^-8^ dilution were added to *P. aeruginosa* PAO1, 100 µL of PSA39 from the 10^-6^ dilution were added to *P. aeruginosa* PA6452, while 100 µL of PSA39 from the 10^-5^ dilution were added to *S. maltophilia* CCV131. The soft agar LB mixed with bacteria and phage was then distributed on LB plates and incubated at 37° overnight. Plaques were imaged the following day. Images are representative of three biological replicates (N = 3).


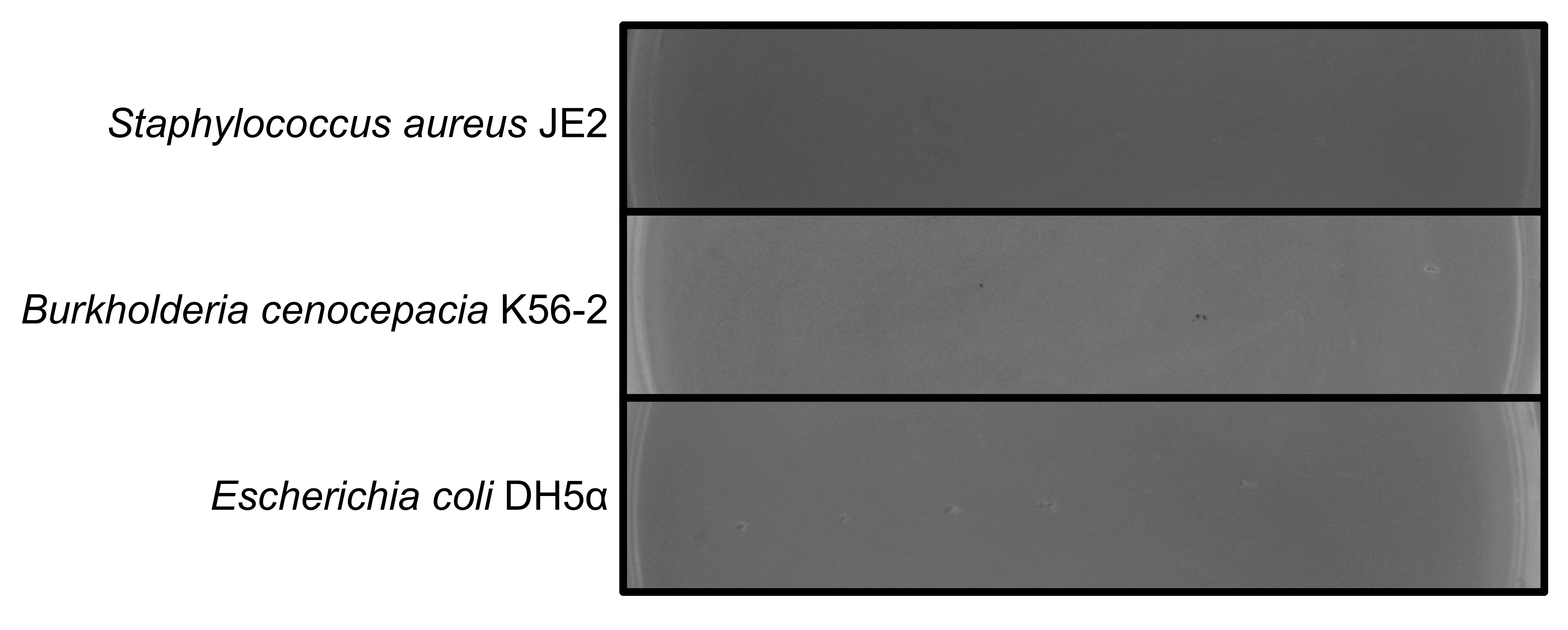


**Supplemental Figure 2. PSA39 does not form plaques on *Staphylococcus aureus*, *Escherichia coli,* or *Burkholderia cenocepacia*.** PSA39 phage lysate was serially diluted and 2 µL of each dilution were spotted on the indicated bacterial strains. Results are representative of three biological replicates (N = 3).


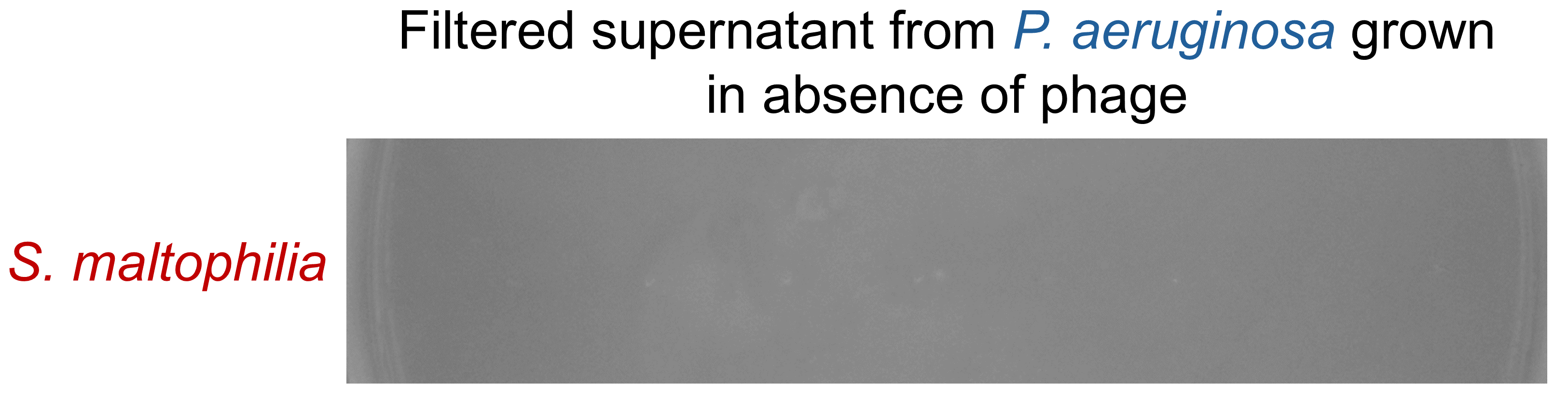


**Supplemental Figure 3. Filtered supernatant from *P. aeruginosa* grown in the absence of PSA39 does not form plaques on *S. maltophilia.*** Filtered supernatant from *P. aeruginosa* grown in the absence of phage was serially diluted and 2 µL of each dilution were spotted *S. maltophilia*. Results are representative of three biological replicates (N = 3).


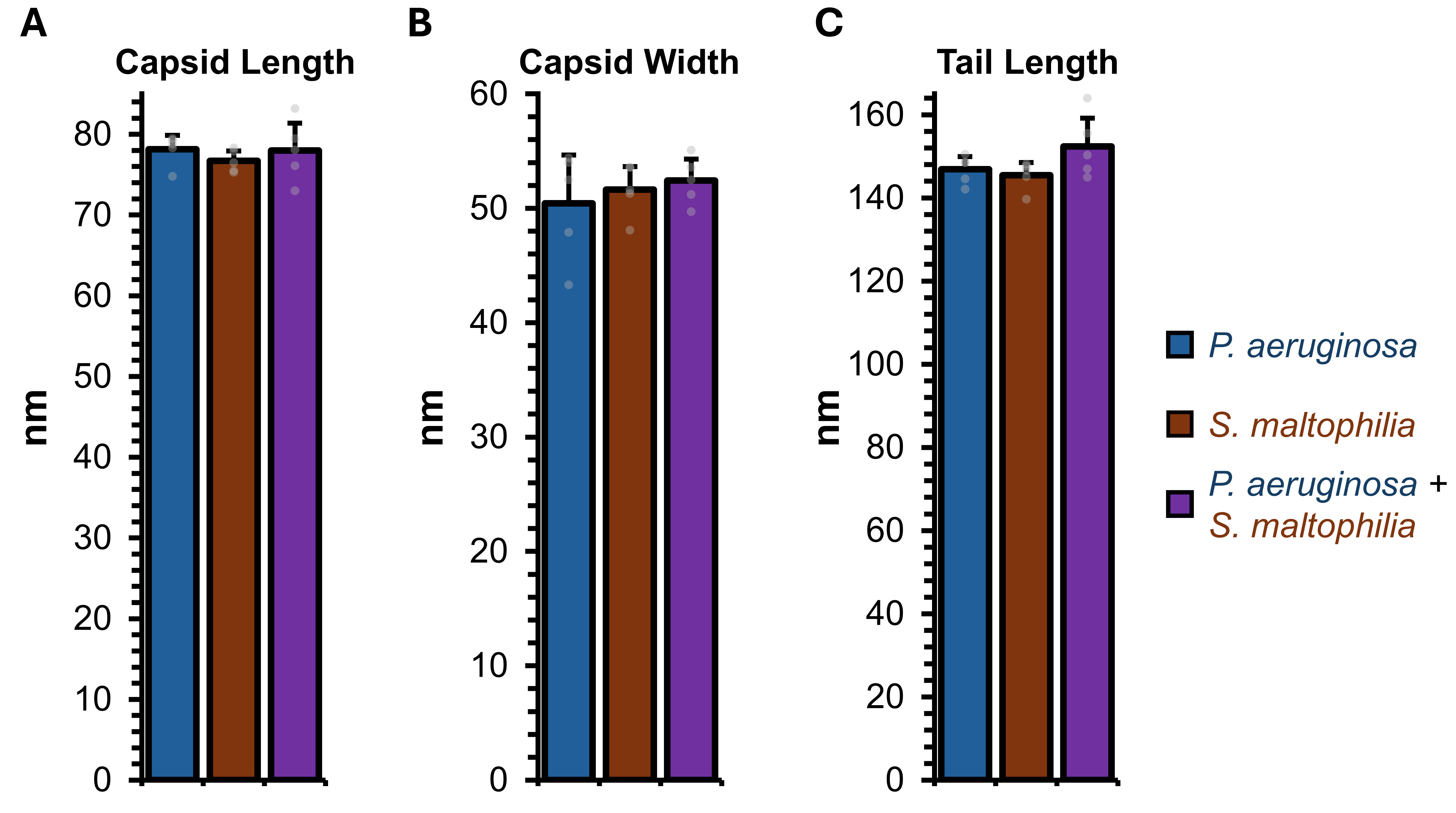


**Supplemental Figure 4. PSA39 capsid length, capsid width, or tail length are independent of the bacterial host*.*** Phage lysates obtained after growth in the presence of *P. aeruginosa* alone*, S. maltophilia* alone*,* or *P. aeruginosa + S. maltophilia* were spotted on carbon-coated copper grids, stained with 2% uranyl acetate, and imaged with a Hitachi HT7800 120 kV Transmission Electron Microscope at 60,000x magnification. Five individual phage particles were used to determine PSA39 capsid length (A), capsid width (B), and tail length measurements (C). Error bars represent standard deviation.


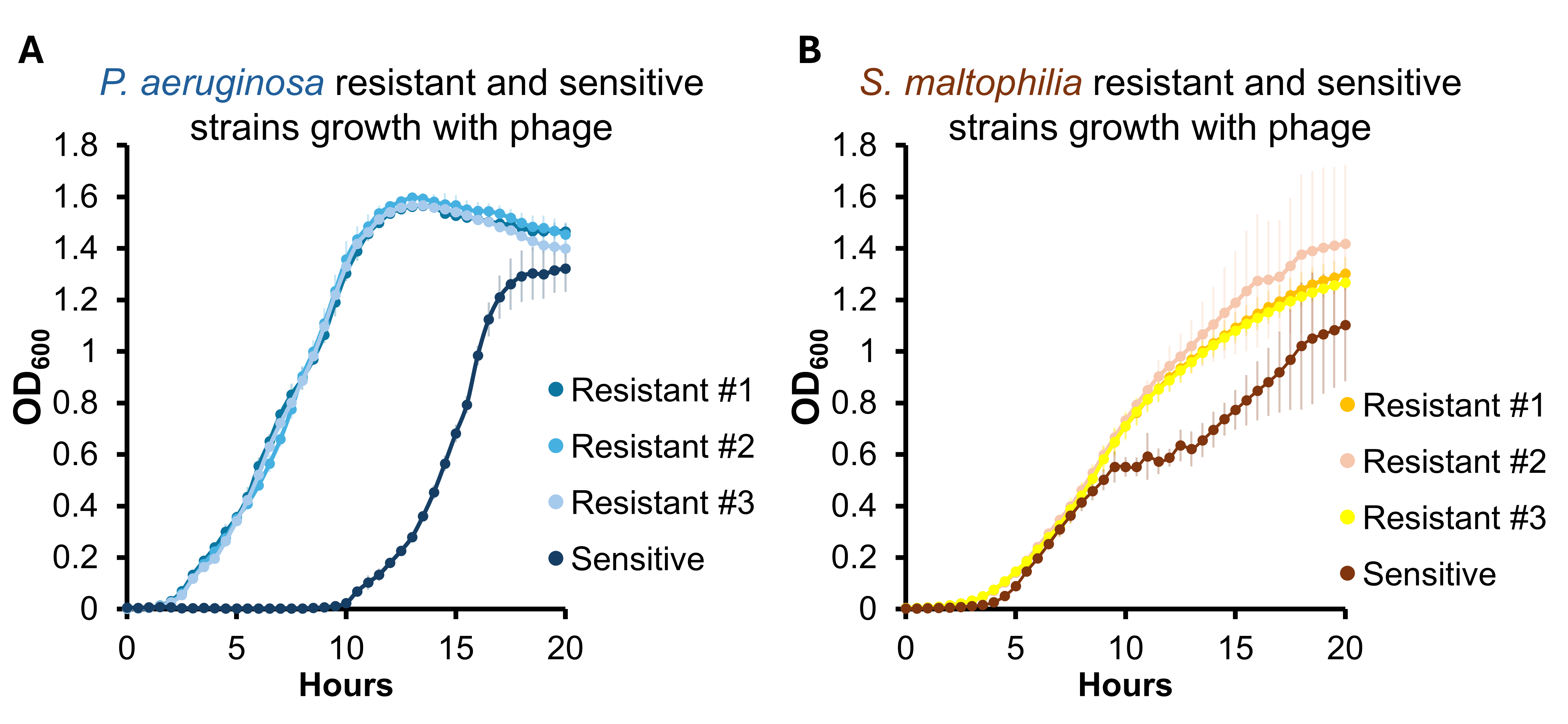


**Supplemental Figure 5. *P. aeruginosa* and *S. maltophilia* strains evolved in the presence of PSA39 are resistant to phage lysis.** Overnight cultures of the indicated resistant or sensitive *P. aeruginosa* and *S. maltophilia* strains were back-diluted 1:50 in fresh liquid LB, grown at 37°C for three hours, and adjusted to an OD_600_ = 0.1. 100 µL of bacteria were added to 3 mL of liquid LB. 5 µL of PSA39 phage (~3 x 10^9^ virions) were added where indicated. 200 µL of each culture were transferred to a 96-well plate and OD_600_ values were recorded at 37°C. Four biological replicates (N = 4) were used to calculate averages. Error bars represent standard deviations.
