# Supplementary material for: A polyvalent phage shapes bacterial dynamics
Source: J Virol. 2026 May 19;100(6):e01363-25. doi: 10.1128/jvi.01363-25 (PMC13288487; doi:10.1128/jvi.01363-25)
Supplement: Supplemental tables — Tables S1 to S6. [file jvi.01363-25-s0002.docx]

**Supplemental Table 1. Bacterial strains used in this study.**

| Strain | Description | Source |
| --- | --- | --- |
| *Pseudomonas aeruginosa* PAO1 | Wild type | Reference strain |
| *Pseudomonas aeruginosa* PAO1 | Isolated after growth in the presence of PSA39 (colony #1) | This study |
| *Pseudomonas aeruginosa* PAO1 | Isolated after growth in the presence of PSA39 (colony #2) | This study |
| *Pseudomonas aeruginosa* PAO1 | Isolated after growth in the presence of PSA39 (colony #3) | This study |
| *Pseudomonas aeruginosa* PAO1 | Isolated after growth in the absence of PSA39 (colony #1) | This study |
| *Pseudomonas aeruginosa* PAO1 | Isolated after growth in the absence of PSA39 (colony #2) | This study |
| *Pseudomonas aeruginosa* PAO1 | Isolated after growth in the absence of PSA39 (colony #3) | This study |
| *Stenotrophomonas maltophilia* CCV131 | Wild type | [1] |
| *Stenotrophomonas maltophilia* CCV131 | Isolated after growth in the presence of PSA39 (colony #1) | This study |
| *Stenotrophomonas maltophilia* CCV131 | Isolated after growth in the presence of PSA39 (colony #2) | This study |
| *Stenotrophomonas maltophilia* CCV131 | Isolated after growth in the presence of PSA39 (colony #3) | This study |
| *Stenotrophomonas maltophilia* CCV131 | Isolated after growth in the absence of PSA39 (colony #1) | This study |
| *Stenotrophomonas maltophilia* CCV131 | Isolated after growth in the absence of PSA39 (colony #2) | This study |
| *Stenotrophomonas maltophilia* CCV131 | Isolated after growth in the absence of PSA39 (colony #3) | This study |
| *Stenotrophomonas maltophilia* CCV119 | Wild type | [1] |
| *Stenotrophomonas maltophilia* CCV123 | Wild type | [1] |
| *Pseudomonas aeruginosa* PA14 | Wild type | [2] |
| *Pseudomonas aeruginosa* PA6487 | Wild type | [3] |
| *Pseudomonas aeruginosa* PA6452 | Wild type | [3] |
| *Staphylococcus aureus* JE2 | Wild type | [4] |
| *Escherichia coli* DH5α | Wild type | Invitrogen |
| *Burkholderia cenocepacia* K56-2 | Wild type | [5] |

**Supplemental Table 2. Type IV pili (T4P) proteins from *P. aeruginosa* PAO1 have limited amino acid sequence identity to their putative *S. maltophilia* CCV131 homologs.**

| *Pseudomonas aeruginosa* PAO1 protein | % query coverage of putative *Stenotrophomonas maltophilia* CCV131 protein homolog | % identity of putative *Stenotrophomonas maltophilia* CCV131 protein homolog |
| --- | --- | --- |
| PilA | 99 | 42.57 |
| PilB | 99 | 53.02 |
| PilC | 100 | 55.94 |
| PilD | 99 | 51.37 |
| PilE | 97 | 39.01 |
| PilF | 60 | 27.45 |
| PilG | 85 | 82.61 |
| PilH | 100 | 57.85 |
| PilI | 99 | 31.25 |
| PilJ | 96 | 47.81 |
| PilK | 90 | 30.32 |
| PilM | 100 | 63.46 |
| PilN | 81 | 41.25 |
| PilO | 93 | 39.70 |
| PilP | 90 | 43.21 |
| PilQ | 76 | 34.96 |
| PilR | 99 | 63.66 |
| PilS | 97 | 37.79 |
| PilT | 99 | 79.06 |
| PilU | 98 | 67.11 |
| PilV | 60 | 29.91 |
| PilW | 26 | 37.14 |
| PilX | - | - |
| PilY1 | 84 | 27.02 |
| PilY2 | - | - |
| PilZ | 92 | 64.86 |
| FimU | 48 | 36.05 |
| FimX | 98 | 34.86 |

**Supplemental Table 3. Results from ANOVA with post-hoc Tukey HSD tests for Figure 3A (Log_10_ recovered *P. aeruginosa* CFUs following monocultures or co-cultures with *S. maltophilia* in the presence or absence of PSA39 phage).** Sm = *Stenotrophomonas maltophilia*, Pa = *Pseudomonas aeruginosa*. Numbers after Sa or Pa indicate the respective percentages of the total inoculum for each bacterial species.

| Comparison | | Tukey p-value |
| --- | --- | --- |
| (Sm0 Pa100 +Phage) | (Sm10 Pa90 +Phage) | 0.000425 |
| (Sm0 Pa100 +Phage) | (Sm50 Pa50 +Phage) | 0.000117 |
| (Sm0 Pa100 +Phage) | (Sm90 Pa10 +Phage) | 0.0000119 |
| (Sm0 Pa100 +Phage) | Sm0 Pa100 NoPhage | 1 |
| (Sm0 Pa100 +Phage) | Sm10 Pa90 NoPhage | 1 |
| (Sm0 Pa100 +Phage) | Sm50 Pa50 NoPhage | 0.419 |
| (Sm0 Pa100 +Phage) | Sm90 Pa10 NoPhage | 0.712 |
| (Sm10 Pa90 +Phage) | (Sm50 Pa50 +Phage) | 0.999 |
| (Sm10 Pa90 +Phage) | (Sm90 Pa10 +Phage) | 0.807 |
| (Sm10 Pa90 +Phage) | Sm0 Pa100 NoPhage | 0.000213 |
| (Sm10 Pa90 +Phage) | Sm10 Pa90 NoPhage | 0.000445 |
| (Sm10 Pa90 +Phage) | Sm50 Pa50 NoPhage | 0.00000265 |
| (Sm10 Pa90 +Phage) | Sm90 Pa10 NoPhage | 0.000008 |
| (Sm50 Pa50 +Phage) | (Sm90 Pa10 +Phage) | 0.976 |
| (Sm50 Pa50 +Phage) | Sm0 Pa100 NoPhage | 0.000059 |
| (Sm50 Pa50 +Phage) | Sm10 Pa90 NoPhage | 0.000122 |
| (Sm50 Pa50 +Phage) | Sm50 Pa50 NoPhage | 0.000000815 |
| (Sm50 Pa50 +Phage) | Sm90 Pa10 NoPhage | 0.00000238 |
| (Sm90 Pa10 +Phage) | Sm0 Pa100 NoPhage | 0.00000619 |
| (Sm90 Pa10 +Phage) | Sm10 Pa90 NoPhage | 0.0000125 |
| (Sm90 Pa10 +Phage) | Sm50 Pa50 NoPhage | 0.000000106 |
| (Sm90 Pa10 +Phage) | Sm90 Pa10 NoPhage | 0.000000291 |
| Sm0 Pa100 NoPhage | Sm10 Pa90 NoPhage | 1 |
| Sm0 Pa100 NoPhage | Sm50 Pa50 NoPhage | 0.59 |
| Sm0 Pa100 NoPhage | Sm90 Pa10 NoPhage | 0.859 |
| Sm10 Pa90 NoPhage | Sm50 Pa50 NoPhage | 0.409 |
| Sm10 Pa90 NoPhage | Sm90 Pa10 NoPhage | 0.701 |
| Sm50 Pa50 NoPhage | Sm90 Pa10 NoPhage | 1 |

**Supplemental Table 4. Results from ANOVA with post-hoc Tukey HSD tests for Figure 3B (Log_10_ recovered *S. maltophilia* CFUs following monocultures or co-cultures with *P. aeruginosa* in the presence or absence of PSA39 phage).** Sm = *Stenotrophomonas maltophilia*, Pa = *Pseudomonas aeruginosa*. Numbers after Sa or Pa indicate the respective percentages of the total inoculum for each bacterial species.

| Comparison | | Tukey p-value |
| --- | --- | --- |
| (Sm10 Pa90 +Phage) | (Sm100 Pa0 +Phage) | 4.65E-07 |
| (Sm10 Pa90 +Phage) | (Sm50 Pa50 +Phage) | 0.285 |
| (Sm10 Pa90 +Phage) | (Sm90 Pa10 +Phage) | 0.0927 |
| (Sm10 Pa90 +Phage) | Sm10 Pa90 NoPhage | 5.93E-09 |
| (Sm10 Pa90 +Phage) | Sm100 Pa0 NoPhage | 2.13E-14 |
| (Sm10 Pa90 +Phage) | Sm50 Pa50 NoPhage | 3.33E-12 |
| (Sm10 Pa90 +Phage) | Sm90 Pa10 NoPhage | 4.95E-14 |
| (Sm100 Pa0 +Phage) | (Sm50 Pa50 +Phage) | 0.000118 |
| (Sm100 Pa0 +Phage) | (Sm90 Pa10 +Phage) | 0.000529 |
| (Sm100 Pa0 +Phage) | Sm10 Pa90 NoPhage | 0.414 |
| (Sm100 Pa0 +Phage) | Sm100 Pa0 NoPhage | 7.44E-12 |
| (Sm100 Pa0 +Phage) | Sm50 Pa50 NoPhage | 0.0000175 |
| (Sm100 Pa0 +Phage) | Sm90 Pa10 NoPhage | 7.55E-09 |
| (Sm50 Pa50 +Phage) | (Sm90 Pa10 +Phage) | 0.998 |
| (Sm50 Pa50 +Phage) | Sm10 Pa90 NoPhage | 8.04E-07 |
| (Sm50 Pa50 +Phage) | Sm100 Pa0 NoPhage | 2.44E-14 |
| (Sm50 Pa50 +Phage) | Sm50 Pa50 NoPhage | 1.42E-10 |
| (Sm50 Pa50 +Phage) | Sm90 Pa10 NoPhage | 6.32E-13 |
| (Sm90 Pa10 +Phage) | Sm10 Pa90 NoPhage | 0.00000318 |
| (Sm90 Pa10 +Phage) | Sm100 Pa0 NoPhage | 2.84E-14 |
| (Sm90 Pa10 +Phage) | Sm50 Pa50 NoPhage | 4.06E-10 |
| (Sm90 Pa10 +Phage) | Sm90 Pa10 NoPhage | 1.42E-12 |
| Sm10 Pa90 NoPhage | Sm100 Pa0 NoPhage | 2.34E-10 |
| Sm10 Pa90 NoPhage | Sm50 Pa50 NoPhage | 0.00319 |
| Sm10 Pa90 NoPhage | Sm90 Pa10 NoPhage | 6.12E-07 |
| Sm100 Pa0 NoPhage | Sm50 Pa50 NoPhage | 0.00000154 |
| Sm100 Pa0 NoPhage | Sm90 Pa10 NoPhage | 0.00875 |
| Sm50 Pa50 NoPhage | Sm90 Pa10 NoPhage | 0.025 |

**Supplemental Table 5. Results from ANOVA with post-hoc Tukey HSD tests for Figure 3C (Log_10_ recovered PFUs plated on *P. aeruginosa* following growth with *P. aeruginosa* and/or *S. maltophilia*).** Sm = *Stenotrophomonas maltophilia*, Pa = *Pseudomonas aeruginosa*. Numbers after Sa or Pa indicate the respective percentages of the total inoculum for each bacterial species.

| Comparison | | Tukey p-value |
| --- | --- | --- |
| Sm0Pa100 | Sm100Pa0 | 6.12E-08 |
| Sm0Pa100 | Sm10Pa90 | 0.000000203 |
| Sm0Pa100 | Sm50Pa50 | 0.000000149 |
| Sm0Pa100 | Sm90Pa10 | 0.000000283 |
| Sm100Pa0 | Sm10Pa90 | 0.854 |
| Sm100Pa0 | Sm50Pa50 | 0.943 |
| Sm100Pa0 | Sm90Pa10 | 0.719 |
| Sm10Pa90 | Sm50Pa50 | 0.999 |
| Sm10Pa90 | Sm90Pa10 | 0.999 |
| Sm50Pa50 | Sm90Pa10 | 0.984 |

**Supplemental Table 6. Results from ANOVA with post-hoc Tukey HSD tests for Figure 3D (Log_10_ recovered PFUs plated on *S. maltophilia* following growth with *P. aeruginosa* and/or *S. maltophilia*).** Sm = *Stenotrophomonas maltophilia*, Pa = *Pseudomonas aeruginosa*. Numbers after Sa or Pa indicate the respective percentages of the total inoculum for each bacterial species.

| Comparison | | Tukey p-value |
| --- | --- | --- |
| Sm0Pa100 | Sm100Pa0 | 1.54E-12 |
| Sm0Pa100 | Sm10Pa90 | 1.95E-12 |
| Sm0Pa100 | Sm50Pa50 | 6.9E-13 |
| Sm0Pa100 | Sm90Pa10 | 1.46E-12 |
| Sm100Pa0 | Sm10Pa90 | 0.999 |
| Sm100Pa0 | Sm50Pa50 | 0.947 |
| Sm100Pa0 | Sm90Pa10 | 1 |
| Sm10Pa90 | Sm50Pa50 | 0.862 |
| Sm10Pa90 | Sm90Pa10 | 0.998 |
| Sm50Pa50 | Sm90Pa10 | 0.961 |

**References**

1. Crisan C V., Pettis ML, Goldberg JB. Antibacterial potential of *Stenotrophomonas maltophilia* complex cystic fibrosis isolates. *mSphere* 2024;**0**:e00335-24. <https://doi.org/10.1128/msphere.00335-24>
2. Rahme LG et al. Common Virulence Factors for Bacterial Pathogenicity in Plants and Animals. *Science (1979)* 1995;**268**:1899–1902. https://doi.org/10.1126/science.7604262

3. Raghuram V, Goldberg JB. Draft genome sequences of eight *Pseudomonas aeruginosa* corneal infection isolates. *Microbiol Resour Announc* 2020;**9**. <https://doi.org/10.1128/MRA.01253-19>
4. Kennedy AD et al. Epidemic community-associated methicillin-resistant *Staphylococcus aureus*: Recent clonal expansion and diversification. *Proceedings of the National Academy of Sciences* 2008;**105**:1327–1332. <https://doi.org/10.1073/pnas.0710217105>
5. Darling P et al. Siderophore production by cystic fibrosis isolates of *Burkholderia cepacia*. *Infect Immun* 1998;**66**:874–877. https://doi.org/10.1128/IAI.66.2.874-877.1998
